# Impact of xylan on field productivity and wood saccharification properties in aspen

**DOI:** 10.3389/fpls.2023.1218302

**Published:** 2023-07-17

**Authors:** Marta Derba-Maceluch, Pramod Sivan, Evgeniy N. Donev, Madhavi Latha Gandla, Zakiya Yassin, Rakhesh Vaasan, Emilia Heinonen, Sanna Andersson, Fariba Amini, Gerhard Scheepers, Ulf Johansson, Francisco J. Vilaplana, Benedicte R. Albrectsen, Magnus Hertzberg, Leif J. Jönsson, Ewa J. Mellerowicz

**Affiliations:** ^1^ Umeå Plant Science Centre, Department of Forest Genetics and Plant Physiology, Swedish University of Agricultural Sciences, Umeå, Sweden; ^2^ Division of Glycoscience, Department of Chemistry, KTH Royal Institute of Technology, AlbaNova University Centre, Stockholm, Sweden; ^3^ Department of Chemistry, Umeå University, Umeå, Sweden; ^4^ Enhet Produktionssystem och Material, RISE Research Institutes of Sweden, Växjö, Sweden; ^5^ Wallenberg Wood Science Centre (WWSC), KTH Royal Institute of Technology, Stockholm, Sweden; ^6^ Umeå Plant Science Centre, Department of Plant Physiology, Umeå University, Umea, Sweden; ^7^ Biology Department, Faculty of Science, Arak University, Arak, Iran; ^8^ Tönnersjöheden Experimental Forest, Swedish University of Agricultural Sciences, Simlångsdalen, Sweden; ^9^ SweTree Technologies AB, Umeå, Sweden

**Keywords:** field trial, GMO, *Populus tremula x tremuloides*, saccharification, salicinoid phenolic glucosides, SilviScan, transgenic trees, xylan

## Abstract

Xylan that comprises roughly 25% of hardwood biomass is undesirable in biorefinery applications involving saccharification and fermentation. Efforts to reduce xylan levels have therefore been made in many species, usually resulting in improved saccharification. However, such modified plants have not yet been tested under field conditions. Here we evaluate the field performance of transgenic hybrid aspen lines with reduced xylan levels and assess their usefulness as short-rotation feedstocks for biorefineries. Three types of transgenic lines were tested in four-year field tests with RNAi constructs targeting either *Populus GT43* clades *B* and *C* (*GT43BC)* corresponding to *Arabidopsis* clades *IRX9* and *IRX14*, respectively, involved in xylan backbone biosynthesis, *GATL1.1* corresponding to *AtGALT1* involved in xylan reducing end sequence biosynthesis, or *ASPR1* encoding an atypical aspartate protease. Their productivity, wood quality traits, and saccharification efficiency were analyzed. The only lines differing significantly from the wild type with respect to growth and biotic stress resistance were the *ASPR1* lines, whose stems were roughly 10% shorter and narrower and leaves showed increased arthropod damage. *GT43BC* lines exhibited no growth advantage in the field despite their superior growth in greenhouse experiments. Wood from the *ASPR1* and *GT43BC* lines had slightly reduced density due to thinner cell walls and, in the case of *ASPR1*, larger cell diameters. The xylan was less extractable by alkali but more hydrolysable by acid, had increased glucuronosylation, and its content was reduced in all three types of transgenic lines. The hemicellulose size distribution in the *GALT1.1* and *ASPR1* lines was skewed towards higher molecular mass compared to the wild type. These results provide experimental evidence that *GATL1.1* functions in xylan biosynthesis and suggest that *ASPR1* may regulate this process. In saccharification without pretreatment, lines of all three constructs provided 8-11% higher average glucose yields than wild-type plants. In saccharification with acid pretreatment, the *GT43BC* construct provided a 10% yield increase on average. The best transgenic lines of each construct are thus predicted to modestly outperform the wild type in terms of glucose yields per hectare. The field evaluation of transgenic xylan-reduced aspen represents an important step towards more productive feedstocks for biorefineries.

## 1 Introduction

Lignocellulose derived from plant cell walls is the largest source of carbon on the Earth (Bar-On et al., 2018) and can be used for sustainable production of energy carriers, green chemicals, and materials. Xylan constitutes approx. 20-30% of lignocellulose in hardwoods, 40-50% in grasses, and 5-15% in softwoods (Scheller and Ulvskov, 2010). It is usually seen as a negative factor in saccharification and fermentation processes because it restricts access to cellulose, cross-links lignin, and forms xylose when hydrolyzed, which is undesirable because xylose is used inefficiently by ethanol-producing yeasts (Donev et al., 2018; Wierzbicki et al., 2019). Xylan in hardwoods and grasses is also an important source of acetic acid in saccharification/fermentation media which is one of the main inhibitors of alcoholic fermentation with yeast (Jönsson and Martín, 2016). Efforts to reduce the xylan content of the lignocellulose or change its structure have therefore been made in many species by suppressing various xylan biosynthetic genes. These measures usually have positive effects on saccharification but may also adversely affect growth (reviewed by Donev et al., 2018, and Wierzbicki et al., 2019). Fortunately, these negative effects can often be avoided or even reversed by fine tuning the degree of gene suppression (Biswal et al., 2015; Ratke et al., 2018). However, field tests of improved xylan-modified plants have only been reported for hybrid aspen (*Populus tremula L. x tremuloides Michx.*) lines with reduced xylan acetylation (Derba-Maceluch et al., 2020; Pramod et al., 2021). Other types of xylan modification have only been tested under greenhouse conditions.

A genetic engineering strategy for xylan reduction in aspen that has performed especially well in greenhouse experiments involves suppressing genes of clades *B* and *C* in the *GT43* family, corresponding to *Arabidopsis* clades *IRX9* and *IRX14*, respectively, which encode proteins belonging to the secondary wall xylan synthase complex (Ratke et al., 2015). These genes are required for the proper function of the xylan UDP-xylosyl transferase complex in *Arabidopsis*, *Populus* and other plant species (Brown et al., 2007; Lee et al., 2007a; Zhou et al., 2007; Lee et al., 2010; Lee et al., 2011; Lee et al., 2012; Ratke et al., 2018). The proteins of these two clades bind the xylan xylosyl transferase encoded by a GT47 member IRX10 (Jensen et al., 2014; Jiang et al., 2016; Zeng et al., 2016) and are essential for both assembly of the complex in the ER and Golgi and its subsequent anchoring to the membrane (Anders et al., 2023). Suppressing these genes in hybrid aspen improved growth and enzymatic saccharification yields (Ratke et al., 2018) and could thus partially alleviate the need for chemical pretreatment of biorefinery feedstocks. However, practical deployment of such modified feedstocks will require field testing of the modified lines.

The biosynthesis of the xylan backbone in dicotyledons and conifers requires the synthesis of a tetrasaccharide known as the reducing-end sequence (RES) that is bound to the backbone at the reducing end (Peña et al., 2007). Mutations in genes responsible for RES biosynthesis reduce xylan levels and secondary wall thickness, producing a so-called “irregular xylem” (irx) phenotype resembling that of xylan synthase mutants (Brown et al., 2007; Peña et al., 2007; Lee et al., 2007b; Brown et al., 2009). At least three genes are known to participate in RES biosynthesis in *Arabidopsis*: *GAUT12/IRX8*, *GATL1*/*PARVUS* and *FRA8/IRX7* (reviewed by Smith et al., 2018). *Populus* has either one or two copies of each of these genes: the copies of *AtGAUT12* are *GAUT12-1* and *GAUT12-2*, which are also known as *GT8D1/GAUT12-A* and *GT8D2/GAUT12-B* (Li et al., 2011; Biswal et al., 2015; Kumar et al., 2019); the copies of *AtGATL1* are *GATL1.1* and *GATL1.2*, also known as *GT8E/GATL1-A* and *GT8F/GATL1-B* (Kong et al., 2009; Lee et al., 2009a; Kumar et al., 2019); and the copy of *AtFRA8* is *GT47C* (Zhou et al., 2006). These *Populus* genes complement the corresponding *Arabidopsis* mutants (Zhou et al., 2006; Kong et al., 2009; Lee et al., 2009a; Biswal et al., 2015) and are thus predicted to function in RES biosynthesis. Suppression of *GAUT12-1* and *GT47C* in *Populus* resulted in decreased xylan content, thinner secondary walls, improved saccharification, and in the case of GAUT12-1 also in increased growth (Lee et al., 2009b; Lee et al., 2011; Li et al., 2011; Biswal et al., 2015), whereas simultaneous suppression of *GAUT12-1* and *GAUT12-2* induced lignification and brittle stems in greenhouse-grown trees (Li et al., 2011). The effects of suppressing *GATL1.1* and *GATL1.2* have not been analyzed to date. Other genes that may participate in xylan backbone biosynthesis include *IRX15* in *Arabidopsis* (Jensen et al., 2011) and genes encoding a germin-like protein and VERNALIZATION PROTEIN 2 in wheat (Jiang et al., 2016). However, the biochemical functions of these genes are currently unknown. Additionally, no xylan backbone or RES sequence biosynthetic mutants or knock-down (KD) lines have been tested under field conditions.

Here we evaluate the field phenotypes of transgenic hybrid aspen lines with suppressed expression of the B and C clades of *GT43* family genes and *GATL1.1* based on a four-year field experiment in Southern Sweden. We also report a novel xylan defect in aspen lines with suppressed expression of *ASPARTIC PROTEASE 1* (*ASPR1*) which was not previously implicated in xylan biosynthesis but was tested here since the gene was highly induced during secondary wall formation in developing wood (Sundell et al., 2017). We evaluate these three genetic modifications in terms of field productivity and biomass saccharification potential to determine their usefulness in efforts to engineer short-rotation biorefinery feedstocks.

## 2 Materials and methods

### 2.1 Plant material

Hybrid aspen (*Populus tremula* L. x *tremuloides* Michx.) clone T89 was used to make transgenic lines (**Table 1**) and as a wild-type (WT) control. Vectors for generation of *35S:ASPR1* and *35S:GATL1.1* lines were based on the plant RNAi vector pK7GWIWG2; cloned cDNA fragments (**Table S1**) were inserted between the vector’s attR1 and attR2 using the GATEWAY system (Karimi et al., 2002). The generation of *WP : GT43BC* lines was described in an earlier publication (Ratke et al., 2018). Transgenic lines were obtained by *Agrobacterium* transformation and clonally replicated *in vitro* as described previously (Ratke et al., 2018). The lines used in the experiment were chosen from 10-20 independent clones based on transgene expression and phenotypes in greenhouse tests. The level of target gene expression in developing wood of selected lines was approx. 50% of WT level for GT43B (Ratke et al., 2018), 45%-57% of WT level for *GATL1.1* and 13%-25% of WT level for *ASPR1* (**Supplemental Figure S1**). The primers for the RT-PCR analysis are listed in **Supplemental Table S2**.

**Table 1:** Lines used in this study.

Construct	*Construct short name*	*Populus* target gene ID	*P. trichocarpa* v3.1 ID	*P. tremula* v2.2 ID	Lines	Ath closest homolog	Ath gene name	Ref
*35S:ASPR1* RNAi	*ASPR1*	*ASPR1*	Potri.019G002100	not available	1B 2A 3A	AT2G03200	ASPR1	Soares et al., 2019
*35S:GATL1.1* RNAi	*GATL1.1*	*GATL1.1*	Potri.014G040300	Potra2n14c26628	1B 1A	AT1G19300	PARVUS, GATL1	Kong et al., 2009; Lee et al., 2009a
*WP: GT43BC RNAi*	*GT43BC*	GT43A	Potri.006G131000	Potra2n6c14160	11 18 20	AT2G37090	IRX9	Ratke et al., 2015; Ratke et al., 2018
		GT43B	Potri.016G086400	Potra2n16c30051				
		GT43C	Potri.007G047500	Potra2n7c16229		AT4G36890, AT5G67230	IRX14, IRX14-L	
		GT43D	Potri.005G141500	Potra2n5c11571				

### 2.2 Field trial establishment and phenotyping

The lines were cultivated in pots for a month at the Umeå Plant Science Centre and then planted with a 3 m spacing in August 2014 in fields located in Laholm community, Sweden (56.42 N, 13.07 E), as described previously (Derba-Maceluch et al., 2020). A permit for their planting was granted by the Swedish Board of Agriculture (DNR.4.6.18-761/14). Two trees per transgenic line and four WT trees were randomly assigned to each of 14 blocks, as shown in **Supplemental Figure S2**. The trial also included other transgenic lines, some of which were previously described (Derba-Maceluch et al., 2020; Pramod et al., 2021). The field was harrowed or mowed twice a year to control weeds.

Plant height was measured with a meter stick, and stem diameter was measured 3 cm from the ground with a caliper four times per year. General damage from biotic and abiotic stress was assessed using the method of Nilsson and Örlander (1999) using a 0-5 scale (no damage - dead tree) while conducting measurements. In July 2017, additional leaf biotic damage assessments were performed as described by Derba-Maceluch et al. (2020); the degree of chewing damage by arthropods (chewing) was quantified as the percentage of chewed leaves in the canopy. Other types of damage were scored in terms of presence (1) and absence (0), including damage caused by aphids, miners, gall-producing organisms, rust (*Melampsora* spp.), venturia (*Venturia* spp.), chlorosis, necrosis, and hypersensitive responses (HR). Chlorophyll content was determined with a CCM-200 plus instrument (Opti-Science, Huston, United States) on six fully developed leaves per tree for 50% of the tallest trees in each line, as previously described (Derba-Maceluch et al., 2020). The same leaves were frozen on dry ice, freeze-dried, and used for analysis of condensed tannins and phenylpropanoids (salicortin, tremulacin, salicin, tremuloidin, salicyloylsalicin, HCH-salicortin, 2′-(E)-, and 2′-(Z)-cinnamoylsalicortin, 2′-acetylsalicin, 2′-acetylsalicortin, acetyl tremulacin, HCH-2′-acetylsalicortin, and HCH-tremulacin, which are known to vary in response to biotic and abiotic stresses in *Populus* leaves (Keefover-Ring et al., 2014). The metabolites were analyzed as described by Derba-Maceluch et al. (2020).

In 2018 the field suffered from exceptional drought (**Supplemental Figure S3**) resulting in the generation of flower buds on some trees. In accordance with permit DNR.4.6.18-761/14 for field trials with transgenic trees, the field was harvested prematurely in September 2018. Stem material was collected from 50% of the tallest trees of each transgenic line and WT, including a five-cm stem segment 10 cm from the ground for SilviScan analyses and a 30-cm stem segment above it for wood chemical analyses. The segments were dried and stored at room temperature before analyses.

### 2.3 SilviScan analyses

SilviScan (CSIRO, Australia) measurements were conducted at RISE Research Institutes of Sweden AB using previously reported procedures (Donev et al., 2023). Briefly, wood characterization of pith to bark samples was performed on three separate measurement units to determine different properties of interest. A video microscope was used to determine the radial distribution of the numbers and sizes of fibers and vessels through image analysis. Subsequently, a high-resolution radial density profile was generated via X-ray absorption scanning. Finally, the microfibril angle at different radial positions was determined by X-ray diffraction scanning.

### 2.4 Stem morphology and branching

To assess differences in branching and growth morphology, trees representing the studied genotypes (N = 14) were photographed in the field before and during bud burst in May and early June of 2017. Images were analyzed using scripts written in the object-oriented programming language Python (Van Rossum and Drake, 2009) using the PlantCV library (Gehan et al., 2017). The method used is available at https://plantcv.readthedocs.io/en/stable/tutorials/vis_tutorial/. The major (*a*) and minor (*b*) axis of the ellipses fitted over the contour of each tree image are obtained, as well as the area of the tree bounding convex hull (*A_ch_*), the tree bounding box length (*L_bb_*) and width (*W_bb_*), and the surface area of the contour of each tree (*A_tree_*), i.e. the number of pixels corresponding to the tree. Other parameters are calculated as follows:


$$\text{Solidity}=\frac{{\text{A}}_{\text{tree}}}{{\text{A}}_{\text{ch}}}$$

$$\text{Elongation}=\frac{{\text{L}}_{\text{bb}}}{{\text{W}}_{\text{bb}}}$$

$$\text{Elipse excentricity}=\sqrt{1-\frac{{\text{b}}^{2}}{{\text{a}}^{2}}}$$

The number of main (primary) and secondary branches was counted manually from the images.

### 2.5 Wood grinding and general chemical characterization

Dry stem segments were cut into pieces roughly 5 cm long and ground in an SM 300 Cutting Mill with a 2 mm sieve (Retsch, Haan, Germany). The rough powder was then sieved using an AS 200 vibratory sieve shaker (Retsch) and divided into the following particle size fractions:< 50, 50–100, 100–500, and > 500 μm. Wood powder of each size fraction from sets of three trees was then pooled. Four such pooled samples (biological replicates) were used for each transgenic line; eight were used for the WT.

Alcohol-insoluble residue (AIR) was obtained from 10 mg of each pooled biological replicate of the 50–100 μm particle size fraction and treated with α-amylase and α-amyloglucosidase (Roche, United States) as described by Gandla et al. (2015). Approx. 50 µg portions of destarched AIR were analyzed by pyrolysis-gas chromatography/mass spectrometry (Py-GC/MS), 500 µg portions were analyzed for matrix sugar composition using an acid methanolysis-trimethylsilyl (TMS) method with two technical replicates, and approx. 3 mg were used for cellulose analysis by the Updegraff method with two technical replicates as described by Pramod et al. (2021).

All four biological replicates of the 50-100 µm sized particle fractions were pooled to obtain one pool representing 12 trees per line for transgenic trees and two pools of 12 trees each for the WT. Each pool was then analyzed in technical duplicates to determine the sugar composition by a two-step sulfuric acid hydrolysis method (Saeman et al., 1954). Aliquots (1 mg) of wood powder were incubated in 125 μL of 72% (w/v) H_2_SO_4_ at room temperature for 3 h, then diluted with 1375 μL of deionized water and incubated at 100°C for 3 h. The resulting hydrolysates were diluted with 0.9 mL of MilliQ water and filtered through a 0.2 mm syringe filter (Chromacol 17-SF-02-N) into a high-performance anion-exchange chromatography - pulsed amperometric detection (HPAEC-PAD) vial for sugar analysis.

### 2.6 Isolation and characterization of hemicelluloses

Hemicelluloses were purified by alkaline extraction (Timell, 1961; Escalante et al., 2012) by incubating 1 g of aspen wood powder of 12 trees, as described above, with 10 mL of a 24% (w/v) aqueous solution of KOH for 24 h at room temperature. The extract was then filtered through a 60 µm filter and neutralized by adding 0.4 volume of glacial acetic acid. Hemicellulose was precipitated with 96% (v/v) ethanol at 4°C overnight, and the precipitate was centrifuged, washed in 80% ethanol, dissolved in distilled water, and freeze-dried to obtain the hemicellulose fraction.

The sugar composition of the alkaline extracts was determined after acidic methanolysis (Bertaud et al., 2002) using 1 mg of dry extract and 1 mL of 2 M HCl in dry methanol, incubating the mixture at 100°C for 5 h. Samples were then neutralized with 200 µL of pyridine, dried under a stream of air, hydrolyzed with 2 M tri-fluoro-acetic acid (TFA) at 120°C for 1 h, air-dried, and dissolved in 1 mL milliQ water. The hydrolysates were analyzed by high-performance anion-exchange chromatography with pulsed amperometric detection (HPAEC-PAD) using a Dionex ICS-6000 instrument (Thermo Fisher Scientific, Stockholm, Sweden) equipped with a Dionex CarboPac PA1 column (4 × 250 mm) at 30°C, applying previously reported eluent gradients (McKee et al., 2016). Monosaccharide composition (monomeric form) was quantified by standard calibration of ten monosaccharides and sugar acids (Ara, Rha, Fuc, Xyl, Man, Gal, Glc, GalA, 4-*O*-MeGlcA and GlcA) with concentrations of 0.005 - 0.1 g/L.

The molar mass of the alkaline extracts was determined by size exclusion chromatography coupled to refractive index and UV-detectors (SECurity 1260, Polymer Standard Services, Mainz, Germany). The freeze-dried extracts were dissolved in dimethyl sulfoxide (DMSO, anhydrous, Sigma-Aldrich) to a concentration of 2 mg/mL with 0.5% w/w LiBr (Anhydrous free-flowing Redi-Dri, Sigma-Aldrich) at 60 °C overnight and filtered through 0.45 μm PTFE syringe filters (VWR). Separation was performed using GRAM Analytical columns of 100 and 10 000 Å (Polymer Standard Services, Mainz, Germany) at a flow rate of 0.5 mL/min at 60 °C. The columns were calibrated using pullulan standards between 345 and 708 000 Da (Polymer Standard Services, Mainz, Germany).

For oligosaccharide mass profiling (OLIMP), the alkali-extracted hemicelluloses were digested using GH30 endo β-(1-4) glucuronoxylanase from *Bacillus subtilis* (St John et al., 2006; St John et al., 2011), kindly provided by Prof. James F. Preston, University of Florida, by incubating 1 mg of dry extract in 20 mM sodium acetate buffer (pH 5.5, 1g/L) with the enzyme (10 U mL^-1^) for 16 h at 37 °C. After incubation, the enzyme was inactivated at 95°C and the mixture was filtered. Oligosaccharides were analyzed by liquid chromatography system (ACQUITY UPLC, Waters, USA) coupled with electron spray tandem mass spectrometer (LC-ESI-MS/MS, Synapt HDMS, Waters, USA) as described by Martínez-Abad et al. (2020). The hydrolysates were desalted with HyperSep Hypercarb cartridges (Thermo Fisher), dissolved in 50% (v/v) acetonitrile and 0.1% (v/v) formic acid, oligosaccharides were purified through a ZORBAX Eclipse Plus C18 column (1.8 µm x 2.1 mm x 50 mm, Agilent Technologies, Santa Clara, CA), and analyzed using positive ion mode in the ESI-MS. The capillary and cone voltages were set to 3 kV and 70 kV, respectively.

### 2.7 Analytical saccharification

Analytical enzymatic saccharification experiments were performed as described previously (Gandla et al., 2021) using 50 mg of dry wood powder (particle size 100-500 µm) from each of four biological replicates per transgenic line and ten biological replicates for WT, with two technical replicates in each case. A biological replicate consisted of pooled stem biomass from three trees. Enzymatic saccharification experiments were performed without pretreatment and with acid pretreatment. The pretreatment and enzymatic hydrolysis conditions were as described previously (Gandla et al., 2021). Briefly, pretreatment was performed in a single-mode microwave system (Initiator Exp, Biotage, Uppsala, Sweden) with sulfuric acid (1% w/w relative to the total weight of the reaction mixture) at 165 ˚C for 10 min, whereas enzymatic hydrolysis was performed in sodium citrate buffer, 50 mM, pH 5.2, using 4 mg (corresponding to approx. 3.5 μL) liquid enzyme preparation (Cellic CTec-2 obtained from Sigma-Aldrich, St. Louis, MO, U.S.A.) per sample. Cellic CTec2 has a reported protein concentration of 182 mg/mL, a cellulase activity of 77 FPU/mL, and a xylanase activity of 160 IU/mL (Qin et al., 2018). Incubation was performed at 45°C and 170 rpm (Ecotron incubator shaker, Infors, Bottmingen, Swizerland) for 72 h. Samples from reaction mixtures were centrifuged at 14 000 rpm for 10 min to separate solid and liquid portions. The glucose production rate (GPR) of the liquid portions after 2 h was determined with a glucometer (Accu-Chek® Aviva; Roche Diagnostics Scandinavia AB, Bromma, Sweden) and monosaccharide sugar yields (Ara, Gal, Glc, Xyl and Man) were determined after 72 h using a Dionex ICS-5000 HPAEC system (Thermo Fisher Scientific) with a set of standard monosaccharide sugar solutions for calibration (Wang et al., 2018).

## 3 Results

### 3.1 Growth of transgenic lines in the field

#### 3.1.1 Survival, plant damage, and general morphology

Young trees belonging to the transgenic lines and WT were planted in the field during the summer of 2014. The mortality up to the summer of 2018 never exceeded one out of 28 trees per line (transgenic lines) or 56 trees (WT) and the survival of the modified trees therefore did not deviate from the survival of WTs (P< 0.05; Fisher’s exact test). The summer of 2018 was exceptionally dry in Southern Sweden (**Supplementary Figure S3**), and the entire field dried out with no surviving trees. Therefore, growth and plant damage data were only considered for the first four growing seasons up to November 2017.

**Figure 1:**
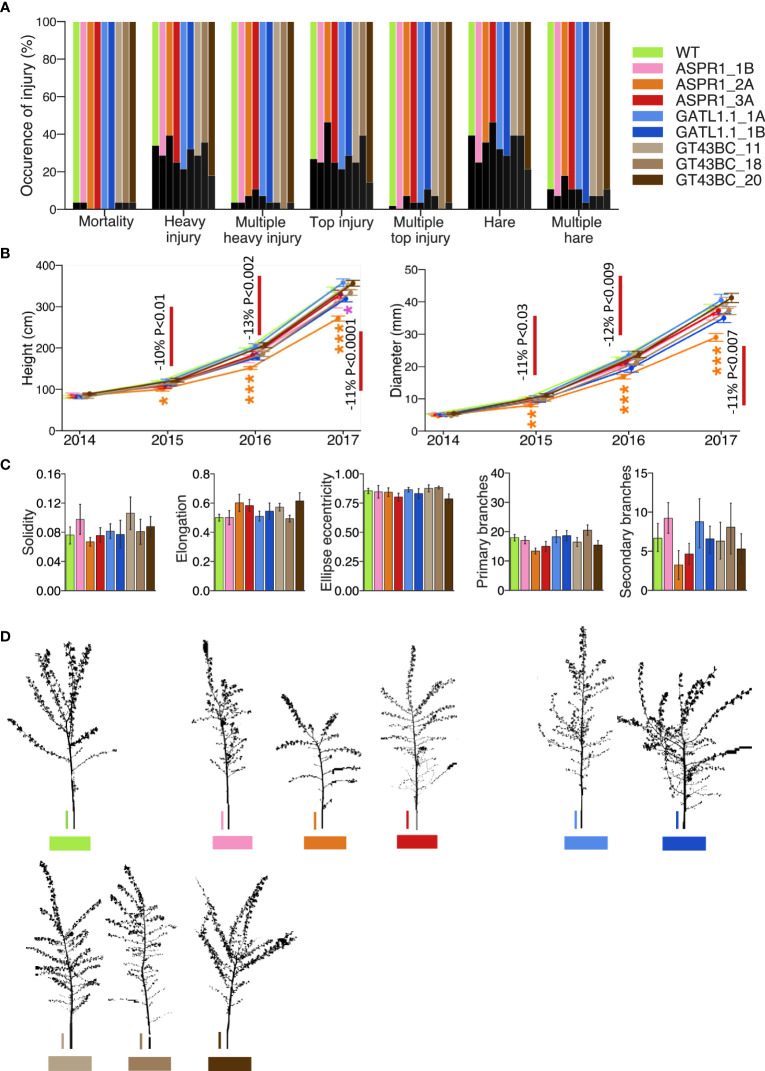
Effect of genotype on plant damage and growth in the field. **(A)** Occurrence of injury evaluated using a general assessment scale. **(B)** Stem height and diameter. **(C)** Stem morphology parameters: solidity, elongation, ellipse eccentricity (as explained in the Material and Methods section), and number of primary and secondary branches per tree based on analyses of contour tree images. **(D)** Representative digitized contour images of trees. Size bar (vertical line) = 30 cm. The analyses in A, B, and C are based on 28, 14, and 7 trees per transgenic line, respectively, or 56, 28, and 14 trees for the wild-type (WT). Data in B-C are means (± *SE*). Asterisks denote significance assessed by Dunnett’s test for comparisons between transgenic lines and WT. (*- P<5%, ** - P<1%, *** - P<0.1%). *P* values indicate the significance of the difference between all lines of a construct and the WT based on contrast analysis.

During the four years of growth, many trees (30%-40%) were damaged by hares, some of them multiple times. This damage tended to occur at specific locations in the field but no significant differences were observed between the lines (**Figure 1A**, **Supplemental Table S3**). Apical shoot damage (with any cause) was observed in 20% to 40% of trees and the frequency did not differ among genotypes. Overall, 30% to 40% of the trees suffered heavy damage (corresponding to a score of 3 on a general damage scale ranging from 0 to 5) and the occurrence of damage was unrelated to genotype. This random damage affected plant growth, so to analyze effects of genotypes on growth we excluded heavily injured plants by selecting the best-growing 50% of the trees in each line.

Height and diameter growth were slightly reduced (by 10-13%) relative to the WT in the *ASPR1* lines, especially in line 2A (**Figure 1B**). The lines of other transgenic constructs did not differ significantly from the WT with respect to either of these variables. The transgenic lines did not differ significantly from the WT with respect to any parameter relating to shoot morphology or tree architecture (**Figures 1C, D**).

Most of the transgenic lines thus exhibited growth similar to the WT and had normal morphology; only the *ASPR1* construct (particularly the *ASPR1_2A* line) showed growth retardation.

#### 3.1.2 Leaf damage, chlorophyll, condensed tannins, and salicinoid glycoside contents

**Figure 2:**
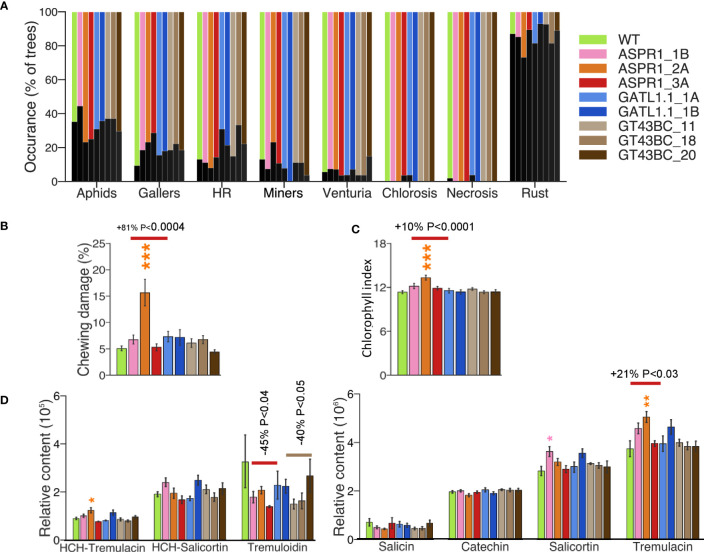
Leaf characteristics and damage. **(A)** Occurrence of injury due to different biotic factors. **(B)** Chewing damage showing % of canopy affected by arthropods; **(C)** Chlorophyll index; **(D)** Leaf content of different salicinoids. The data in B-D are means (± *SE*); *n*=28 transgenic and 56 wild-type (WT) trees **(A, B)**, 13 transgenic and 25 WT trees **(C)** or 4 transgenic or WT trees **(D)**. Asterisks denote significance assessed by Dunnett’s test for comparisons between transgenic lines and WT. (* *P*< 5%, ** *P<* 1%, *** *P<* 0.1%). *P* values indicate the significance of the difference between all lines of a construct and the WT based on contrast analysis.

The leaves of field-grown trees were analyzed during the summer of 2017. Biotic damage levels were relatively low in that summer: of the damage types evaluated based on presence, only rust occurred frequently (**Figure 2A**). Leaf damage caused by chewing arthropods was also common, and the percentage of canopy that was affected by arthropods was significantly higher in the *ASPR1_2A* line than in the WT and other lines (**Figure 2B**).

With just one exception, there were no significant differences between genotypes with respect to leaf chlorophyll content: the *ASPR1_2A* line had a higher chlorophyll index than the WT (**Figure 2C**). Several phenolic compounds were identified in the leaves; the *ASPR1* and *GT43BC* lines exhibited reduced levels of tremuloidin relative to the WT, and the *ASPR1* line also had elevated levels of tremulacin (**Figure 2D**). No consistent deviations from WT levels were observed for other phenolic compounds (**Supplementary Table S4**).

### 3.2 Wood anatomy and SilviScan traits

**Figure 3:**
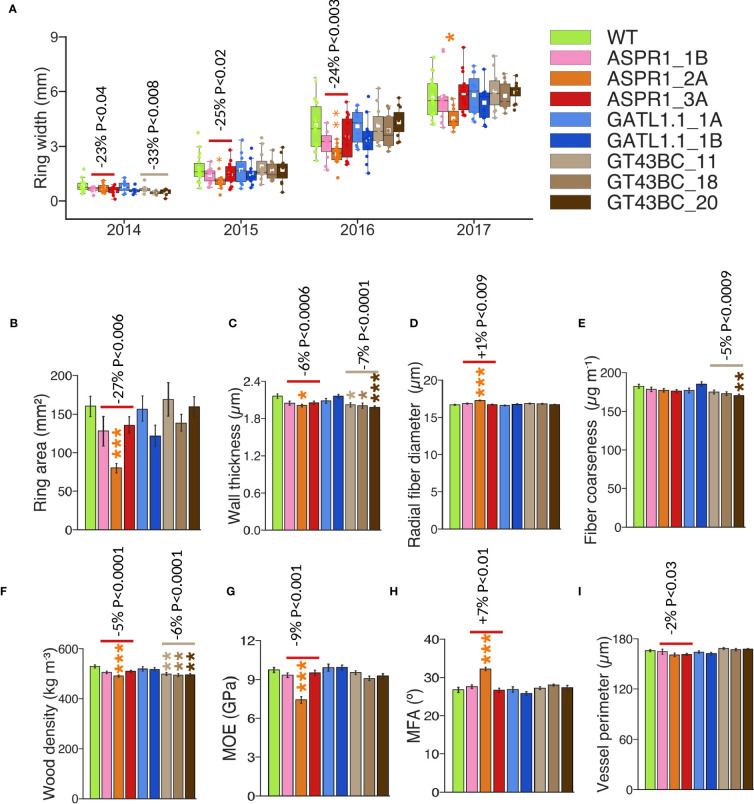
Effect of genotype on wood anatomy and wood properties analyzed by SilviScan. **(A)** Width of the annual rings; **(B)** Mean ring area for years 2015-2017; **(C)** Mean fiber cell wall thickness. **(D)** Mean radial fiber diameter; **(E)** Mean fiber coarseness; The data are based on the best-performing 50% of trees for each line and represent means (± *SE*) of *n*=12 or 18 trees for transgenic lines and the wild-type (WT). Data for **(C–H)** are means for 2015-2017 growth rings (ring area weighted). Data in **(A–F)** are based on 25 µm resolution optical scanning. Data in **(G, H)** are based on 5 mm resolution X-ray diffraction scanning. Data in **(I)** are based on 2 mm resolution optical scanning. *P* values indicate the significance of the difference between all lines of a construct and the WT based on contrast analysis.

Wood of transgenic and WT trees was analyzed using a SilviScan instrument. The annual ring width initially decreased in the *ASPR1* and *GT43BC* lines, but by the year 2017 this behavior was only observed in the *ASPR1_2A* line (**Figure 3A**). Average wood traits were calculated for the 2015, 2016, and 2017 rings; the first ring was excluded because it was not always present in the dissected-out samples. Average annual ring area was reduced in *ASPR1* lines (**Figure 3B**) and cell wall thickness was slightly reduced in the *ASPR1* and *GT43BC* lines. Consequently, the wood density in these lines was slightly lower (by 5% and 6%, respectively) than in the wild type, and the *GT43BC* lines also exhibited reduced fiber coarseness (by 5%) (**Figures 3C, E, F**). The *ASPR1* lines exhibited a slight increase in fiber and vessel size (**Figures 3D, I**), which may have contributed to their low wood density. The *ASPR1* lines also exhibited a slight reduction in modulus of elasticity (MOE; by 9%) and a slight increase in cellulose microfibril angle (MFA; by 7%). Overall, these changes were very modest; the SilviScan wood traits were only slightly affected in the *ASPR1* and *GT43BC* lines and not at all in the *GATL1.1* lines.

### 3.3 Wood chemistry

**Figure 4:**
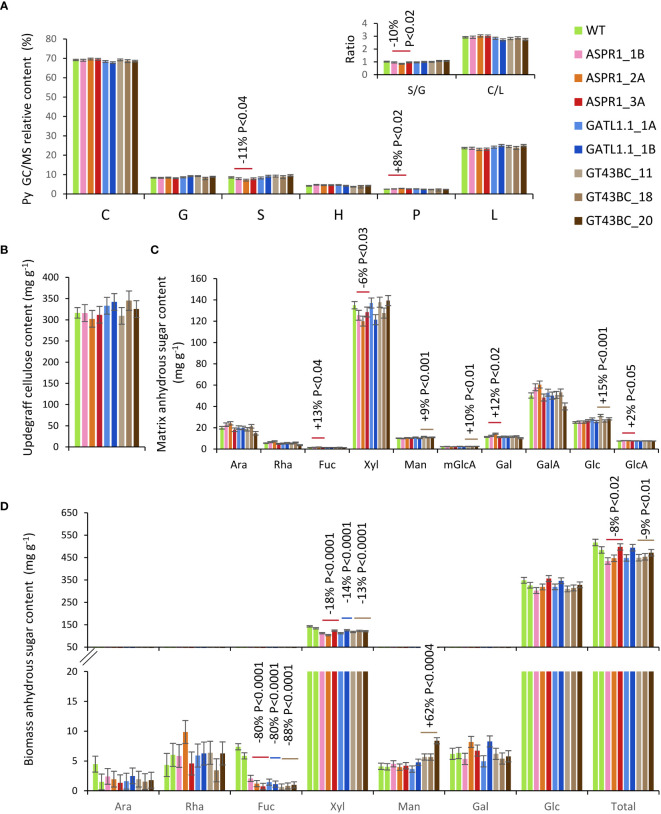
Effect of genotype on stem biomass chemical composition. **(A)** Relative peak integrals for compounds detected by pyrolysis-gas chromatography/mass spectrometry (Py-GC/MS). C-carbohydrates; G – guaiacyl units; S-syringyl units; H- *p*-hydroxyphenol units; P – phenolics: L – total lignin (G+S+H+P); **(B)** Updegraff cellulose content; **(C)** Acid methanolysis – trimethylsilyl (TMS) matrix sugar content of biomass; **(D)** Biomass sugar content based on two-step hydrolysis. The data are means (± *SE*) of *n* = 4 or 8 biological replicates for transgenic lines and wild-type (WT) plants, respectively **(A–C)** or 2 technical replicates of 12 pooled trees **(D)**. *P* values indicate the significance of the difference between all lines of a construct and the WT based on contrast analysis.

Pyrolysis-GC/MS analysis of stem biomass revealed that the biomass composition of the transgenic lines did not differ significantly from that of the WT with only one exception: the S-lignin content and S/G ratio of the *ASPR1* lines were slightly reduced and their phenolic content was increased (**Figure 4A**). None of the transgenic lines differed significantly from the WT with respect to Updegraff cellulose content (**Figure 4B**). Matrix sugar content recovered by the acid methanolysis-TMS amounted to roughly 40% of the total biomass in all analyzed samples. Compared to the WT, *ASPR1* lines had reduced levels of Xyl and increased levels of Fuc, Gal and GlcA (by -6%, +13%, +12% and +2%, respectively), while *GT43BC* lines had increased levels of Man, mGlcA and Glc (by 9%, 10% and 15%, respectively) (**Figure 4C**).

When the biomass sugar composition was analyzed using two-step hydrolysis with sulfuric acid, approx. 50% of the biomass weight was recovered, with Glc and Xyl being main sugars (**Figure 4D**). All transgenic lines exhibited reduced levels of Xyl (by 13% to 18%) relative to the WT. In *GT43BC* construct lines, this effect was partially compensated by an increase in the content of Man, but the content of Glc was unaffected in agreement with the unaffected content of crystalline cellulose. All transgenic lines also exhibited strongly reduced levels of Fuc (80% to 88%), which was a minor component.

**Figure 5:**
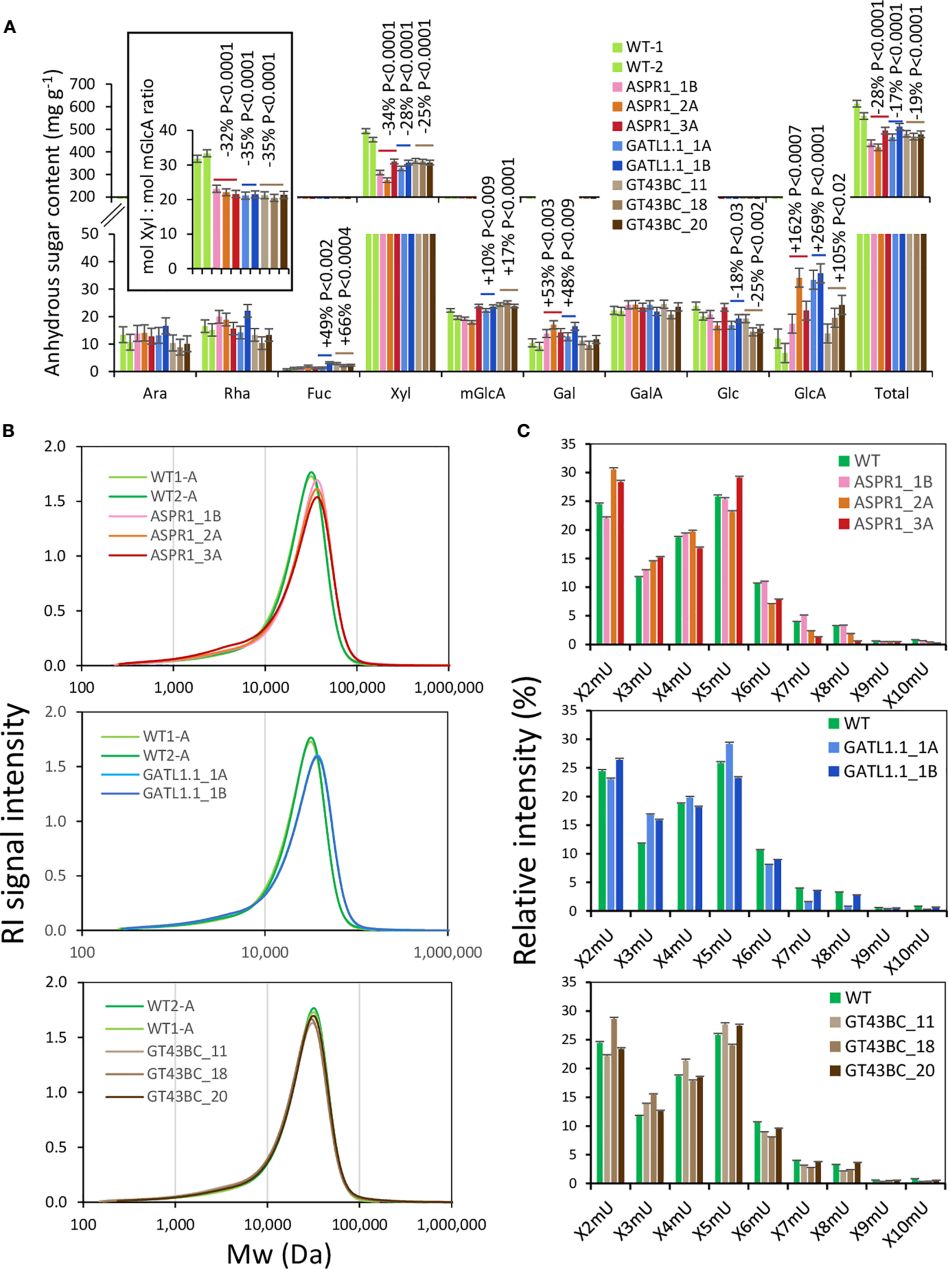
Effect of genotype on alkali-extracted hemicelluloses. **(A)** Hemicellulose sugar composition analysis by acid methanolysis - trifluoroacetic acid (TFA) high-performance anion-exchange chromatography with pulsed amperometric detection (HPAEC-PAD). The inset shows a decrease in the Xyl to mGlcA ratio in transgenic lines. *P* values indicate the significance of the difference between all lines of a construct and the wild-type (WT) based on contrast analysis. **(B)** Size exclusion analysis of hemicelluloses. **(C)** OLIMP analysis of alkali-extracted hemicelluloses. X-xylose unit; mU – mGlcA unit. The data in **A** and **C** are means (± *SE*) of *n* = 2 technical replicates representing 12 pooled trees.

Hemicelluloses were analyzed by extracting biomass samples with a 24% KOH solution. After precipitation, this led to the recovery of 60% of the biomass weight for WT samples, but only around 50% for transgenic lines (**Figure 5A**) in the form of anhydrous sugars. This difference occurred because the Xyl content of the transgenic lines was 25-34% lower than in the WT. The loss of Xyl was not accompanied by any proportional loss of mGlcA; the molar ratio of Xyl to mGlcA was reduced by over 30% relative to the WT in all transgenic lines, indicating increased xylan glucuronosylation. Other changes observed in the hemicellulose sugars of the transgenic lines included strongly increased levels of GlcA (between 2 and 3.5 fold increases) in all transgenic lines, reduced levels of Glc and increased levels of Fuc in *GATL1.1* and *GT43BC* lines, and increased levels of Gal in *ASRP1* and *GATL1.1* lines.

The molecular weight distribution of hemicellulose was only slightly affected in transgenic lines (**Figure 5B**). All transgenic lines had a slightly increased abundance of low molecular weight polymers between 10^3^ and 10^4^ Da, which formed a shoulder of the main peak, and the *ASPR1* and *GATL1.1* construct lines also showed a shift of the main peak towards a higher molecular weight.

The profiles of oligosaccharides released by digestion with xylanase GH30, which attacks the glycosidic bond in the backbone one Xyl unit away from the mGlcA side chain towards the non-reducing end (St John et al., 2006; St John et al., 2011; Martínez-Abad et al., 2018), provided information about changes in the xylan glucuronosylation pattern in transgenic lines. The most abundant oligosaccharides were X2mU and X5mU, indicating glucuronosylation at every second and every fifth Xyl unit (**Figure 5C**). X3mU, X4mU, and X6mU oligosaccharides were also relatively abundant. The transgenic lines tended to have fewer X6mU units and more X3mU units than the WT. Certain lines also showed increased occurrence of X2mU units. Overall, these changes indicate a denser glucuronosylation pattern in transgenic lines compared to the WT.

### 3.4 Saccharification

**Figure 6:**
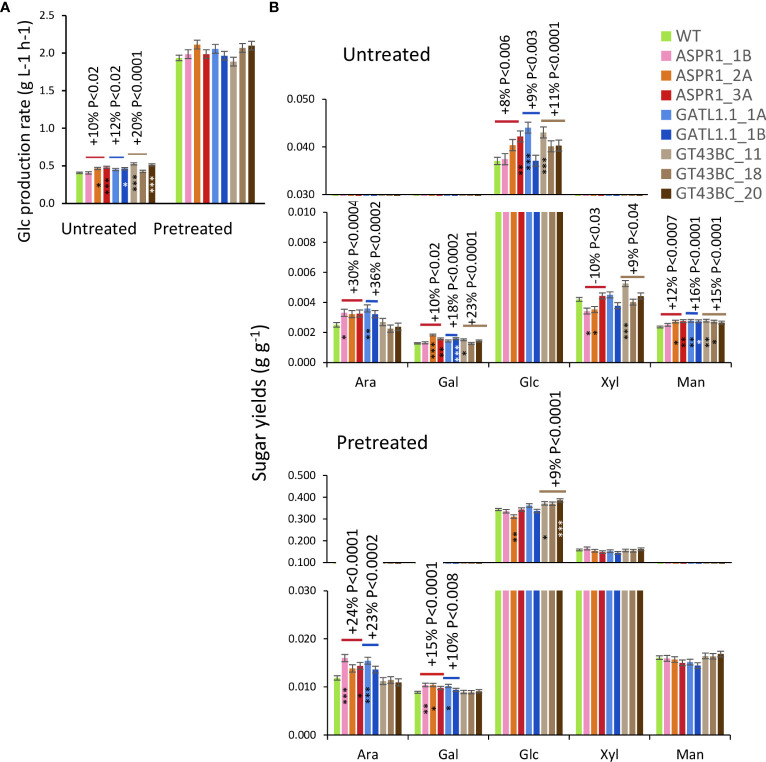
Effect of genotype on saccharification. **(A)** Glc production rates. **(B)** Sugar yields (in hydrous form) of untreated biomass and total yields after acid and enzymatic hydrolysis. Data are means (± *SE*), *n* = 4 or 10 biological replicates for each transgenic line and wild-type (WT), respectively. *P* values indicate the significance of the difference between all lines of a construct and the WT based on contrast analysis. Asterisks indicate means of transgenic lines differing significantly from the WT (Dunnett’s test, * – *P*<5%, ** – *P*<1%, *** – *P*< 0.1%.

Biomass from stems was subjected to enzymatic saccharification without pretreatment (untreated) yielding approx. 5% of biomass recovered as sugars, or to enzymatic saccharification after acid pretreatment (pretreated) with the recovery above 50% of biomass weight (**Figure 6**). All transgenic lines showed increased Glc production rates and Glc yields in saccharification without pretreatment. Matrix sugars were not targeted by the enzymatic mixture used here but all of them other than Xyl were released more readily from the transgenic lines than from the WT.

Biomass of transgenic and WT trees gave similar yields of Xyl, Man and Glc in acid pretreatment liquid (**Supplementary Figure S4**). However, the *ASPR1* and *GATL1.1* lines gave increased yields of Ara and Gal relative to the WT. Total yields of Glc in saccharification with acid pretreatment were increased from biomass of *GT43BC* construct lines (by 9% on average) even though Glc production rates did not differ significantly from WT for any transgenic line. The total yields of sugars other than Ara and Gal (which were released during acid hydrolysis) were unaffected by acid pretreatment.

## 4 Discussion

### 4.1 All three constructs reduced xylan levels and altered its structure in field-grown transgenic lines

We evaluated the effects of downregulating three different classes of enzymes in field-grown hybrid aspen and found that they all affected xylan structure. *GT43BC* construct lines had reduced expression of two clades of glycosyl transferases from the *GT43* family (Ratke et al., 2018). Suppression of these genes by 50% was previously shown to weaken xylan backbone signals relative to RES signals (indicating a chain length reduction of 5-10%) without affecting the acid methanolysis - TMS xylosyl content of cell walls under greenhouse conditions (Ratke et al., 2018). Stronger suppression (80%) of *GT43B* strongly reduced the xylosyl content determined by alditol acetates method (Lee et al., 2011). Under field conditions, *GT43BC* lines exhibited no reduction in acid methanolysis - TMS xylosyl content (**Figure 4C**) but did exhibit reduced Xyl unit content in HPAEC-PAD analyses of biomass subjected to two-step sulfuric acid hydrolysis and in methanolysed alkali-extracted hemicelluloses (**Figure 4D**, **Figure 5A**). Because the three sugar content analysis methods used in this work involve different depolymerization and derivatization conditions and used either destarched AIR or milled wood as their substrate, variable results would be expected. The two-step acid hydrolysis should be considered as providing a full spectrum of all sugars present in cell wall polysaccharides and it probably recovered most of the Xyl from transgenic and WT biomass. It has revealed a 13% reduction in Xyl content in transgenic lines bearing the *GT43BC* construct. A greater reduction of 25% in Xyl content was observed in alkaline extracts of these transgenic lines, suggesting that they have a larger fraction of alkali-insoluble xylan than the WT. Conversely, since similar amounts of Xyl were hydrolyzed by mild acid treatment in both transgenic and WT samples (**Figure 4D**, **Supplemental Figure S4**), it appears that the WT samples had relatively more acid-labile xylan. Thus, the three methods used for Xyl determination provided information about genotypic differences in xylan extractability/accessibility as well as its content. The *ASPR1* and *GATL1.1* transgenic lines showed similar trends in Xyl levels in all analyses performed here as the *GT43BC* lines, indicating that all three constructs reduced xylan levels, increased xylan resistance to alkaline extraction, and reduced xylan resistance to mild acid hydrolysis.

For all three groups of transgenic lines, the Xyl:mGlcA ratio in the hemicellulose fractions was clearly lower than in the WT and the pattern of distributions of xylo-oligosaccharides (XOS) released by GH30 indicated a reduced frequency of widely-spaced glucurosylation and an increased abundance of more tightly spaced glucuronosylation (**Figures 5A, C**). Thus, the xylan of all analyzed transgenic lines had increased glucuronosylation level. The xylan fraction with evenly spaced mGlcA is the dominant xylan domain and is capable of binding the hydrophilic surface of cellulose microfibrils, while the fraction with unevenly spaced mGlcA is a minor domain that cannot bind this surface (Bromley et al., 2013; Grantham et al., 2017). These two xylan domains are generated in the Golgi by the GUX1 and GUX2 clades of the GUX protein family, respectively (Bromley et al., 2013; Lyczakowski et al., 2021). None of the transgenes used in this study affected specifically any of these domains, rather they caused an overall increase in glucuronosylation (**Figure 5C**). This could have been driven by increased pools of UDP-Xyl in the Golgi caused by reduced rate of xylan backbone biosynthesis, creating a product-inhibition feedback for the UDP-GlcA to UDP-Xyl conversion and thus increasing the available substrate UDP-GlcA pool for GUX1 and GUX2 enzymes.

Inhibition of xylan backbone biosynthesis could also affect the backbone chain length. Xylan synthase complex mutants reportedly reduced the degree of polymerization (DP) of xylan (Peña et al., 2007; Brown et al., 2009; Lee et al., 2010; Wu et al., 2010), and a previous quantification of RES and backbone Xyl signals revealed a small reduction of backbone chain length in transgenic *GT43BC* hybrid aspen (Ratke et al., 2018). However, SEC analysis of alkaline-extracted hemicellulose from the same lines grown under field conditions revealed no substantial change in the xylan mass distribution (**Figure 5B**). This probably reflects limitations of the SEC method rather than an effect of growth conditions because the increased xylan glucuronosylation in the transgenic lines could mask small reductions in the xylan backbone DP. On the other hand, RES mutants were reported to have a broader distribution of xylan molecular weights than xylan synthase impaired plants, with some xylan having increased DP (Peña et al., 2007; Brown et al., 2009). In accordance with these reports, we observed a shift in the main hemicellulose peak to higher molecular weights and a slight enhancement of a low molecular weight shoulder in lines with suppressed *GATL1.1*, which probably functions in RES biosynthesis (Kong et al., 2009). Our analysis of hemicellulose sugar composition in these lines (**Figure 5A**) supports the involvement of *Populus GATL1.1* in xylan biosynthesis. Moreover, the altered hemicellulose molecular mass profiles of these lines (**Figure 5B**) are consistent with the changes observed in RES mutants, suggesting that *GATL1.1* plays a role in xylan RES biosynthesis.

### 4.2 Function of *ASPR1*

*ASPR1* belongs to a large family of atypical aspartic proteases and nucellins. Its clade includes eight genes in *P. trichocarpa* and ten in *Arabidopsis* (**Supplemental Figure S5**). *P. tremula* has only four gene models for this family in the v 2.2 assembly, and the model for the current RNAi construct is missing. It has been suggested that the *AtASPR1* cleavage site is located downstream of a leucine residue, but its specificity is unknown (Soares et al., 2019). The two other genes of this clade, *AtECS1* and *AtECS2*, act during fertilization to prevent polytubey (Yu et al., 2021). These peptidases are secreted by fertilized ovule and hydrolyze the Cys-rich pollen tube attractant peptides LURE1.1-LURE1.5. *AtASPR1* is expressed strongly in the root tips, lateral root primordia, pollen grains, and developing vascular tissue (Soares et al., 2019). Its overexpression in *Arabidopsis* inhibited lateral root formation and affected proteins related to biotic and abiotic stresses but its function in vascular development has not been studied. *PtASPR1* is strongly induced in developing xylem during secondary wall formation (Sundell et al., 2017) suggesting involvement in secondary wall biosynthesis. The reduced Xyl content in lines with suppressed *ASPR1* expression compared to WT was detectable in methanolized biomass (**Figure 4A**), fully hydrolyzed biomass (**Figure 4B**) and in alkali-extractable hemicelluloses (**Figure 5A**) and the levels of reduction in these lines were comparable to those seen in lines with suppressed expression of known xylan biosynthesis genes, implicating *PtASPR1* in xylan biosynthesis. Moreover, the XOS profiles, the reduced Xyl to mGlcA ratio, and the molecular weight distribution of the hemicellulose fraction from the *ASPR1* lines closely resembled those of *GATL1.1* construct lines (**Figures 5B, C**). These observations suggest that *ASPR1* may regulate xylan RES biosynthesis.

### 4.3 Evaluation of trait stability under greenhouse and field conditions

There were both drastic (Taniguchi et al., 2012; Dominguez et al., 2021) and modest (Derba-Maceluch et al., 2020; Pramod et al., 2021) differences in the performance of the transgenic trees when comparing greenhouse and field conditions. Because the *GT43BC* lines tested in the field in this work were previously studied under greenhouse conditions (Ratke et al., 2018), it was possible to evaluate the stability of several of their traits. Some traits showed high stability under both growth conditions – for example, the transgenic lines exhibited reduced secondary wall thickness and wood density, increased acid methanolysis - TMS Man contents, and unchanged acid methanolysis - TMS Xyl contents as well as improved biomass saccharification properties in enzymatic saccharification without pretreatment in the field (**Figures 3C, F**, **4C**, **6A, B**) and the greenhouse (Ratke et al., 2018). However, other traits were not reproduced under field conditions. For example, the S-lignin content and S/G ratio of field-grown transgenic trees did not differ from those in the WT but were reduced under greenhouse conditions. The highly improved growth phenotype observed in the greenhouse experiments (Ratke et al., 2018) was also not reproduced in the field (**Figures 1B**, **2A**). The improvements in Glc yields during saccharification without pretreatment were also lower than expected based on the greenhouse experiments (16% and 8% versus 27% and 40% for line 11 and 18, respectively) (**Figure 6B**). However, the field- grown lines exhibited a 9% improvement in saccharification yields after acid pretreatment that was not observed in the greenhouse. Thus, some traits of transgenic trees were stable regardless of the growth conditions while others changed in the field.

### 4.4 Evaluation of transgenic lines as potential biorefinery feedstocks from short-rotation plantations

In the field, the *GATL1.1* and *GT43BC* construct lines did not differ from the WT with respect to any productivity parameters relating to growth, morphology, biotic damage, or the chlorophyll index. However, *ASPR1* lines exhibited mildly reduced growth (an 11% reduction in height and diameter) as well as a higher chlorophyll index and more extensive chewing damage (**Figures 1**, **2**). These lines also showed the most pronounced reduction in Xyl content (**Figures 3C, D**, **5A**). Levels of foliar tannins and salicinoid glucosides were generally unaltered in the transgenic lines, but the *GT43BC* and *ASPR1* construct lines exhibited reduced levels of tremuloidin and the *ASPR1* lines exhibited elevated levels of tremulacin (**Figure 2D**, **Supplementary Table S3**). These differences in salicinoid phenolic glycosides could potentially have affected the plants’ relationships with antagonists (Cole et al., 2021), but we found no evidence of any direct relationship. The wood anatomical traits of the transgenic lines were generally unaltered except for small (5%-7%) reductions in cell wall thickness and wood density in *GT43BC* and *ASPR1* lines as well as a reduced MOE in *ASPR1* construct lines, which was probably due to their increased MFA (**Figures 3C, E-H**). A substantial decrease in secondary wall thickness was also reported for xylan biosynthetic mutants and transgenic lines with reduced expression of xylan biosynthetic genes in which xylan content was decreased (Zhou et al., 2006; Brown et al., 2007; Lee et al., 2007b; Brown et al., 2009; Lee et al., 2011; Wu et al., 2009; Wu et al., 2010; Li et al., 2011). This decrease was previously attributed directly to their reduced xylan content. However, an analysis of transcriptomic changes in developing wood of *GT43BC* construct lines revealed that the suppression of the *GT43* clade *B* and *C* genes caused a downregulation of the entire secondary wall biosynthetic program (Ratke et al., 2018). Moreover, the relatively slight reduction in Xyl content observed in transgenic lines, considering that xylan constitutes only one fourth of cell wall biomass, is unlikely to induce noticeable change in cell wall thickness. The reduced cell wall thickness seen in *ASPR1* construct lines in this study, similar to cell wall thickness reductions in *GT43BC* lines, supports the hypothesis that xylan deficiency in secondary walls may affect secondary wall biosynthesis more generally.

**Figure 7:**
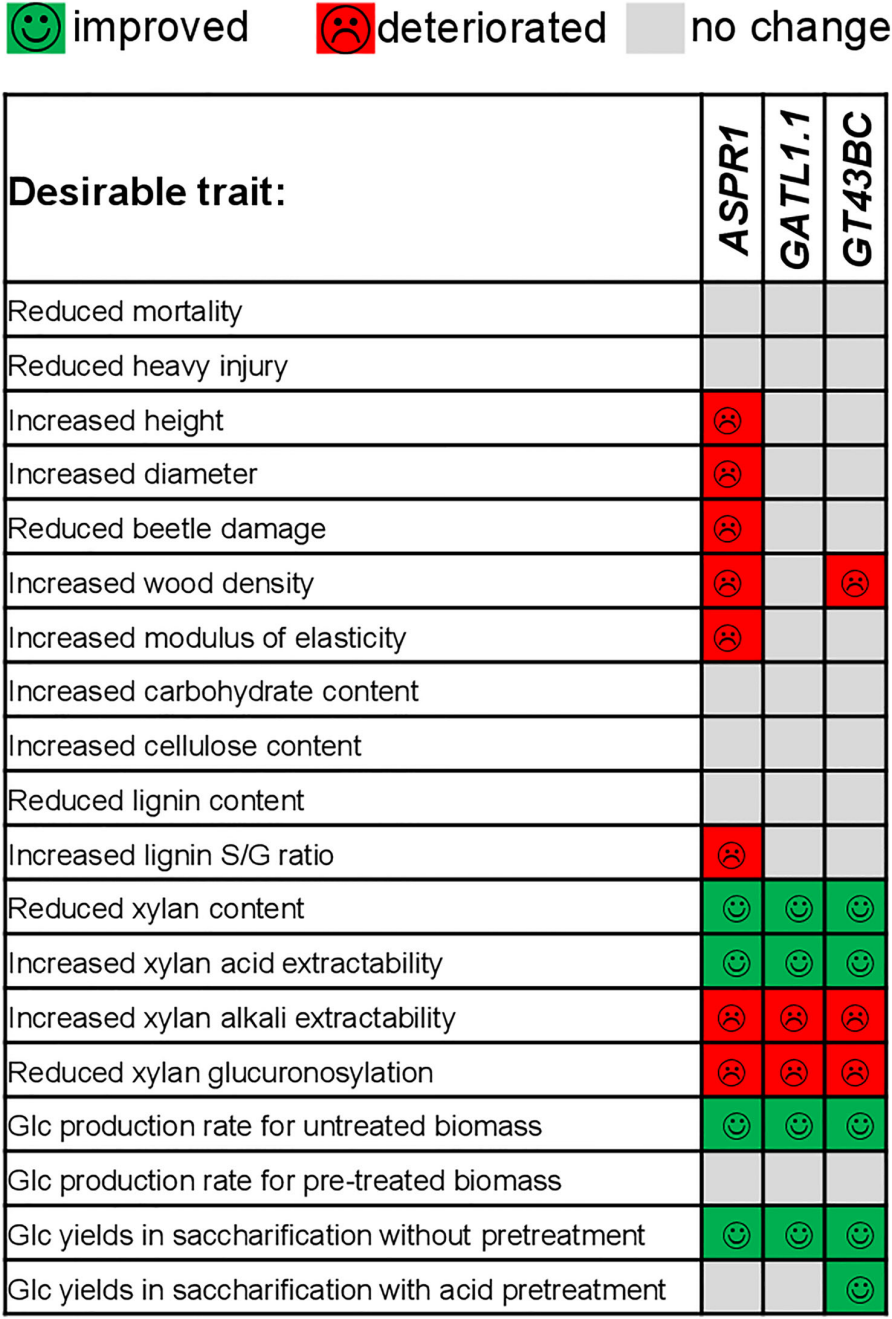
Overview of most important traits that were improved or deteriorated in the field-grown transgenic lines compared to wild-type.

Considering the potential of the studied lines as biorefinery feedstocks, we found that all constructs improved Glc yields in saccharification without pretreatment (**Figures 6A, B**). The overview of changes in most relevant productivity and saccharification traits of transgenic lines is shown in **Figure 7**. The best lines of each transgene, *ASPR1_3A*, *GATL1.1_1A*, and *GT43BC_11*, provided Glc yield increases of 13%, 19% and 16%, respectively. However, only *GT43BC* construct lines provided improved Glc yields of saccharification with acid pretreatment, with the best line giving a 15% increase. As neither cellulose content not matrix Glc content (except for *GT43BC_11*) were increased in these lines, this indicated that the genetic change that resulted in decreased xylan content in these lines lead to an altered cell wall architecture and higher accessibility of saccharification enzymes to substrates. In the case of *GT43BC* lines, the increased accessibility was also observed after acid pretreatment, which is of particular interest considering the presence of the pretreatment step in current technologies. Because these lines exhibit normal growth but some have slightly lower wood density than WT plants, these higher Glc yields should result in modestly improved total Glc gains per hectare.

## Data availability statement

The original contributions presented in the study are included in the article/**Supplementary Material**. Further inquiries can be directed to the corresponding author.

## Author contributions

MD-M overviewed field trial setting up and phenotyping in 2017. PS carried out wood grinding and chemical analyses with help of RV and EH and supervision of FV and EM. ED carried out statistical analyses, data plotting and prepared the figures. MG and LJ were responsible for pretreatment and saccharification analyses. ZY and GS were responsible for SilviScan analyses. UJ was responsible for field management, operation and phenotyping. FA and BA were responsible for biotic stress estimation in 2017 and leaf phenotyping. MH produced and selected transgenic lines for field testing, EM coordinated the study, ensured the funding and wrote the paper with contributions from all the authors. All authors contributed to the article and approved the submitted version.

## Supplementary material

Supplementary Figure 1 Expression levels of targeted genes in developing wood tissues.
