# Supplementary material for: Impact of xylan on field productivity and wood saccharification properties in aspen
Source: Front Plant Sci. 2023 Jul 17;14:1218302. doi: 10.3389/fpls.2023.1218302 (PMC10389764; doi:10.3389/fpls.2023.1218302)
Supplement: Supplementary Figure 1 — Expression levels of targeted genes in developing wood tissues. [file DataSheet_1.docx]

Supplementary Material

**For: Impact of xylan on field productivity and wood saccharification properties in aspen**

Marta Derba-Maceluch, Pramod Sivan, Evgeniy N. Donev, Madhavi Latha Gandla, Zakiya Yassin, Rakhesh Vaasan, Emilia Heinonen, Sanna Andersson, Fariba Amini, Gerhard Scheepers, Ulf Johansson, Francisco J. Vilaplana, Benedicte R. Albrectsen, Magnus Hertzberg, Leif J. Jönsson, Ewa J. Mellerowicz^*^

# Supplementary Material:

**Figure S1** Expression levels of targeted genes in developing wood tissues.

**Figure S2** Field layout and design.

**Figure S3** Meteorological data from the field site 2014-2018.

**Figure S4** Sugar yields of acid pretreatment of saccharification.

**Figure S5** Phylogenetic tree of *ASPR1* gene family in *A. thaliana*, *P. trichocarpa* and *P. tremula*.

1. **(b)**

**Supplementary Figure S1.** **Target gene expression levels in developing wood tissues determined by RT-PCR. (a)** *GATL1.1* **(b)** *ASPR1.* Primers used for the analysis are listed in Supplementary Table S2*. UBQL - Potri.005G198700* was used for normalization. Data are means (± *SE*), n = 3 or 4 biological replicates for *GATL1.1* and *ASPR1*, respectively.


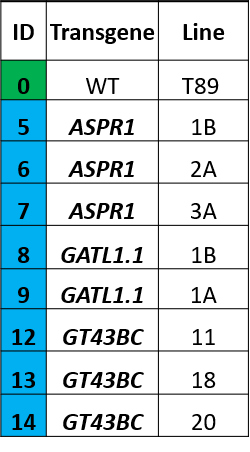

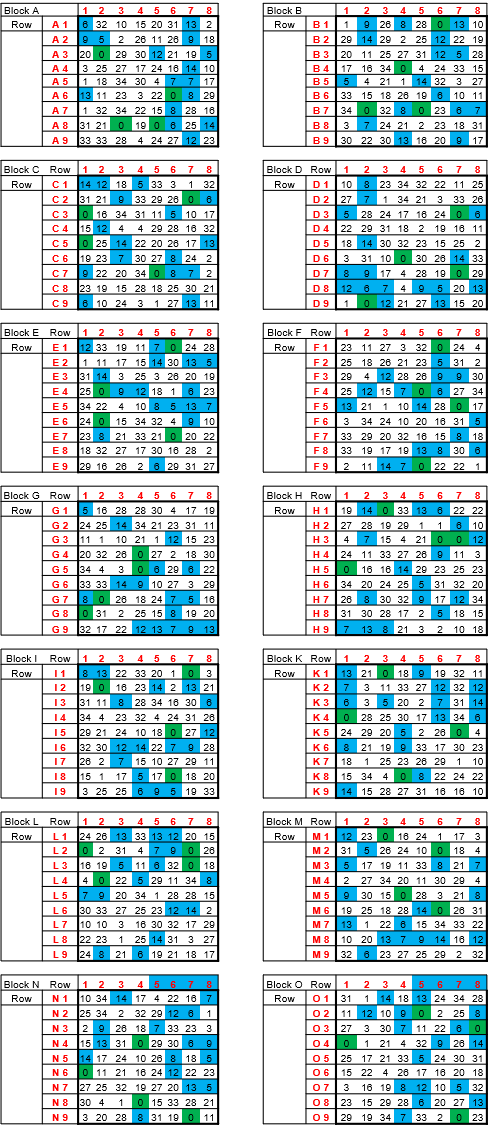


**(b)**

A B

C D

E F

G H

I K

L M

N O

**(c)**

**(a)**

**Supplementary Figure 2.** **Field layout and design**. (**a**) Overview of the field showing localization of the blocks A-O. Lines tested in this study (**b**) and their localization in the blocks (**c**).

**Supplementary Figure S3.** **Meteorological data from the field site 2014-2018.**  (**A**) Monthly temperature. (**B**) Monthly humidity. (**C**) Monthly precipitation.

**Supplementary Figure S4. Sugar yields of acid pretreatment of wood powder in saccharification with acid pretreatment.** Data are means (± *SE*), n = 4 or 10 biological replicates for each transgenic line and WT, respectively. Hydrous sugar forms were quantified. P values indicate significance of the difference for all lines of a construct from WT based on contrast analysis. Asterisks indicate means of transgenic lines significantly different from WT (Dunnett’s test, * P<5%, ** – P<1%, *** – P< 0.1%. .

**Supplementary Figure S5.** **Phylogenetic tree of *ASPR1* gene family in *A. thaliana*, *P. trichocarpa* and *P. tremula*.** The tree was based on amino acid alignment and phylogenetic tree analysis using UPMGA at http:phylogeny.fr. The bootstrap values are based on 500 replicates. The expression information for *A. thaliana* was retrieved from www:arabidopsis.org

**
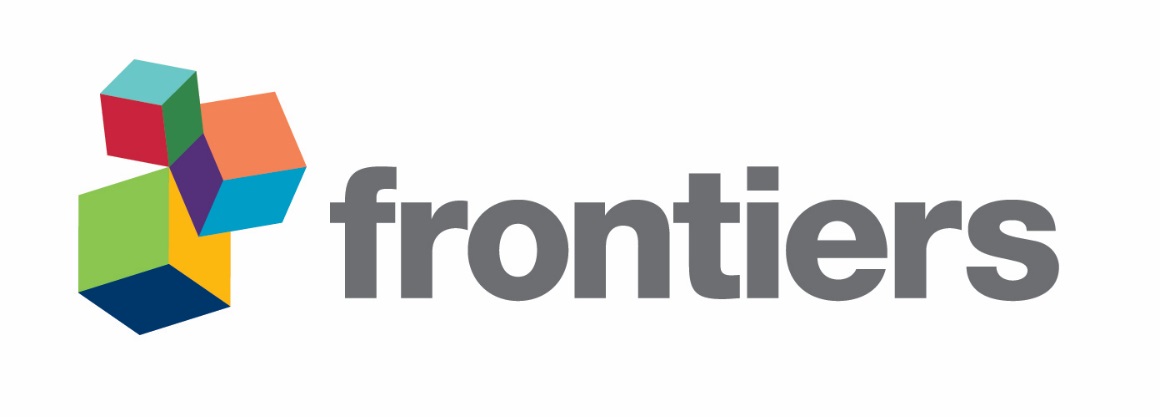
**
